# Kinetics of Enteric Pathogen Quantity During Acute Diarrhea in Children in Resource-Limited Settings

**DOI:** 10.1093/ofid/ofag309

**Published:** 2026-05-26

**Authors:** William J Lain, Sarah E Elwood, Jie Liu, Elizabeth T Rogawski McQuade, Gagandeep Kang, Margaret N Kosek, Aldo A M Lima, Pascal O Bessong, Amidou Samie, Rashidul Haque, Estomih R Mduma, Jose Paulo Leite, Ladaporn Bodhidatta, Najeeha T Iqbal, Nicola Page, Ireen Kiwelu, Zulfiqar A Bhutta, Tahmeed Ahmed, Eric R Houpt, James A Platts-Mills

**Affiliations:** Division of Infectious Diseases and International Health, University of Virginia School of Medicine, Charlottesville, Virginia, USA; Division of Infectious Diseases and International Health, University of Virginia School of Medicine, Charlottesville, Virginia, USA; School of Public Health, Qingdao University, Qingdao, China; Division of Infectious Diseases and International Health, University of Virginia School of Medicine, Charlottesville, Virginia, USA; Department of Public Health Sciences, University of Virginia, Charlottesville, Virginia, USA; Division of Gastrointestinal Sciences, Christian Medical College, Vellore, India; Division of Infectious Diseases and International Health, University of Virginia School of Medicine, Charlottesville, Virginia, USA; Asociación Benéfica PRISMA, Iquitos, Peru; Center of Biomedicine and Department of Physiology and Pharmacology, Faculty of Medicine, Federal University of Ceara, Fortaleza, Brazil; Department of Microbiology, University of Venda, Thohoyandou, South Africa; Department of Microbiology, University of Venda, Thohoyandou, South Africa; Infectious Disease Division, International Centre for Diarrhoeal Disease Research, Bangladesh, Dhaka, Bangladesh; Haydom Global Health Research Centre, Haydom, Tanzania; Fundação Oswaldo Cruz, Rio de Janeiro, Brazil; Department of Enteric Diseases, Armed Forces Research Institute of Medical Sciences, Bangkok, Thailand; Department of Paediatrics and Child Health, Aga Khan University, Karachi, Pakistan; Centre for Enteric Diseases, National Institute for Communicable Diseases, Johannesburg, South Africa; Biotechnology Research Laboratory, Kilimanjaro Clinical Research Institute, Moshi, Tanzania; Department of Paediatrics and Child Health, Aga Khan University, Karachi, Pakistan; Infectious Disease Division, International Centre for Diarrhoeal Disease Research, Bangladesh, Dhaka, Bangladesh; Division of Infectious Diseases and International Health, University of Virginia School of Medicine, Charlottesville, Virginia, USA; Division of Infectious Diseases and International Health, University of Virginia School of Medicine, Charlottesville, Virginia, USA

**Keywords:** diarrhea, pathogen quantity, qPCR

## Abstract

Pathogen quantity kinetics during childhood diarrhea have implications for etiology ascertainment. Using a multisite cohort, we modeled quantitative PCR quantity by symptom duration. Rotavirus had the largest log reduction in quantity by day 7 (−1.98; 95% confidence interval: −2.88, −1.08), followed by *Shigella* (−0.87; −1.46, −.28) and heat-stable toxin-producing *Escherichia coli* (−0.78; −1.57, .02). While peak quantity was robust to host factors, kinetics were not. For example, malnourished children had a minimal decline in *Cryptosporidium* quantity over the first 7 days of illness. Pathogen quantity, etiology attribution, and non-PCR test sensitivity all peaked early in illness. Diarrheal studies should collect samples early to reduce bias in etiology identification.

## BACKGROUND

Diarrhea remains an important syndrome in resource-limited settings [1, 2], and estimating pathogen-specific disease burden is critical. We have shown that quantitative PCR (qPCR) can improve population-level etiology attribution [1]. Additionally, for studies where identifying the cause of each episode is needed [3, 4], we have developed quantitative cutoffs to assign etiology [5, 6]. Just as postdiarrheal shedding duration is highly pathogen-specific [7], pathogen quantity kinetics during episodes may be similarly variable. Small studies have provided limited data for a few pathogens [8–10], but pathogen kinetics have not been systematically evaluated.

Previously, we described the duration of postdiarrheal detection of common diarrheal pathogens from the Etiology, Risk Factors, and Interactions of Enteric Infections and Malnutrition and the Consequences for Child Health and Development study (MAL-ED) [7, 11]. Here, we use the same study to estimate pathogen quantity as a function of symptom duration at the time of sample collection, evaluate modifying factors, and discuss implications for etiology assignment in these settings.

## METHODS

### Study Design, Data Collection, and Derived Variables

The MAL-ED study has been previously described [11, 12]. Between November 2009 and February 2012, children were enrolled after birth in 8 countries. Fieldworkers attempted to visit children twice weekly to monitor illnesses, antibiotic use, and vaccine administration. Diarrhea was defined by maternal report of at least 3 loose stools in 24 hours or visible blood. Upon identification of diarrhea by a field worker, a sample was collected. Stool samples could be collected up to 48 hours prior to diarrhea onset, during illness, or up to 48 hours diarrhea cessation.

An episode was considered a first infection if the pathogen had not been detected with a cycle threshold (*Ct*) <30 more than 30 days prior to symptom onset, consistent with a natural immunity analysis from the MAL-ED study [13]. Day 0 was defined as the day of symptom onset. A child was considered malnourished if their length-for-age *Z* score was less than −2. Locations received approval from their institutional ethics review boards. Informed consent was obtained from the parent or guardian of each child.

### Stool Testing

The methods for both the original microbiologic work-up and qPCR re-analysis have been described [11, 12, 14]. In the original work-up, *Shigella* was detected by stool culture. Heat-stable toxin-producing enterotoxigenic *Escherichia coli* (ST-ETEC) was detected by endpoint PCR using 5 pooled lactose-fermenting colonies from stool culture. Enzyme immunoassay (EIA) was used to detect *Cryptosporidium* (TechLab, Blacksburg, VA, USA), rotavirus, human adenovirus (including nonenteric adenoviruses), and astrovirus (ProSpecT, Remel, Lenexa, KS, USA). Real-time PCR was used to detect norovirus GII, while sapovirus detection was not included. All available diarrheal and monthly nondiarrheal stool samples from children with complete follow-up to 24 months of age were re-tested using customized TaqMan Array Cards (Thermo Fisher, Carlsbad, CA, USA).

### Data Analysis

For the top 8 diarrhea causes [11], we identified all episodes with a sample collected between 48 hours before and 8 days after symptom onset in which the pathogen was detected by qPCR. To increase the likelihood that the pathogen was causative, we required that no alternative pathogen was assigned as etiologic by detection with an episode-specific attributable fraction >0.5 [11]. For each pathogen, we fit a linear regression model, with pathogen quantity as the outcome and a linear and quadratic term for symptom day at stool collection, child age, location, severity score, presence of blood, and quantity of the other pathogens as predictors. We made a marginal prediction at the mean of pathogen quantity as a function of the symptom day at sample collection using the *ggeffects* package in R [15]. To estimate kinetics by child age, presence of malnutrition, and history of prior infection, we added interaction terms between both the linear and quadratic terms for the symptom day and the stratification variable. A *P* value <.20 for either interaction term was considered consistent with heterogeneity, meaning that pathogen kinetics were statistically significantly different between the strata. For all estimates, we reported log 10 reductions in quantity, where a 1-log reduction is equivalent to a *Ct* increase of log_2_10 (∼3.322 cycles). To estimate the change in pathogen quantity over time, we identified the peak day for each pathogen in the nonstratified model for each pathogen and calculated the change in predicted log quantity from that peak to symptom day 7. We estimated quantity differences between strata as marginal effects from the regression models.

To estimate the symptom duration-specific sensitivity of non-PCR diagnostics used in the original MAL-ED analysis in comparison to a PCR gold standard, we fit a logistic regression model with an outcome of conventional test positivity, and linear and quadratic terms for the day of sample collection, child age, site, severity score, and maternal report of blood as predictors. We then produced marginal predictions of original test positivity by symptom day. Finally, for each pathogen, we estimated population-level attribution for all samples collected on a given symptom day by summing the pathogen-specific AFes and dividing by the number of samples collected on that symptom day.

## RESULTS

Of 44 427 stool samples collected, 6692 were from diarrheal episodes, of which 6632 (99.1%) were collected up to 48 hours prior to or up to 8 days after symptom onset. Across the 8 pathogens of interest, there were between 664 and 1671 detections during diarrheal episodes, of which between 370 and 972 did not have an alternative etiology and were thus included in the pathogen-specific kinetics models (Table 1). The median day of sample collection was day 1 (the second day of symptoms) for all pathogens except norovirus GII (day 2), and the median duration of diarrhea was 3–4 days.

**Table 1.** Pathogen Detection From Diarrheal Episodes in MAL-ED and Characteristics of Diarrheal Episodes

	Adenovirus 40/41	Astrovirus	Norovirus GII^a^	Rotavirus	Sapovirus	*Shigella*	ST-ETEC	*Cryptosporidium*
Pathogen detected	1671	1540	1220	879	1635	1207	1461	664
Pathogen detected and no other etiology	932	911	851	587	972	750	752	370
Symptom day at sample collection	1 (0–2)	1 (0–2)	2 (1–3)	1 (1–2)	1 (0–3)	1 (0–2)	1 (0–2)	1 (0–2)
Duration of diarrheal episode	3 (2–5)	3 (2–6)	4 (2–6)	4 (2–5)	4 (2–6)	3 (2–6)	3 (2–5.25)	3 (2–6)
Age at diarrheal onset	9 (6–15)	10 (5–16)	9 (6–13)	9 (5–14)	12 (8–16)	16 (12–20)	12 (8–17)	14 (9–19)
0–11 m	569 (61.1%)	519 (57.0%)	574 (67.5%)	371 (63.2%)	475 (48.9%)	186 (24.8%)	351 (46.7%)	134 (36.2%)
12–23 m	363 (38.9%)	392 (43.0%)	277 (32.5%)	216 (36.8%)	497 (51.1%)	564 (75.2%)	401 (53.3%)	236 (63.8%)
Malnourished	278 (29.8%)	254 (27.9%)	206 (24.2%)	145 (24.7%)	286 (29.4%)	264 (35.2%)	245 (32.6%)	107 (28.9%)
Not malnourished	601 (64.5%)	560 (61.5%)	547 (64.3%)	404 (68.8%)	581 (59.8%)	429 (57.2%)	443 (58.9%)	228 (61.6%)
First infection	534 (57.3%)	559 (61.4%)	461 (54.2%)	468 (79.7%)	505 (52.0%)	448 (59.7%)	357 (47.5%)	254 (68.6%)
Repeat infection	398 (42.7%)	352 (38.6%)	390 (45.8%)	119 (20.3%)	467 (48.0%)	302 (40.3%)	395 (52.5%)	116 (31.4%)
Maternal report of blood in stool	42 (4.5%)	31 (3.4%)	27 (3.2%)	19 (3.2%)	38 (3.9%)	102 (13.6%)	30 (4.0%)	12 (3.2%)
Positive conventional test	85 (9.1%)	212 (23.3%)	NA	263 (44.8%)	NA	103 (13.7%)	168 (22.3%)	170 (45.9%)
Pathogen detected, no other etiology assigned, and pathogen assigned as etiology	213	214	224	407	364	468	228	77

N (%) are shown for dichotomous variables, and median (interquartile range [IQR]) are shown for continuous variables. Malnourished was defined as a length-for-age *Z* score <−2.

Abbreviations: NA, not available; ST-ETEC, heat-stable toxin-producing *Escherichia coli*.

^a^In the original microbiologic work-up, norovirus GII was detected by qPCR and sapovirus was not detected; thus, no conventional test result was available.

**Figure 1.**
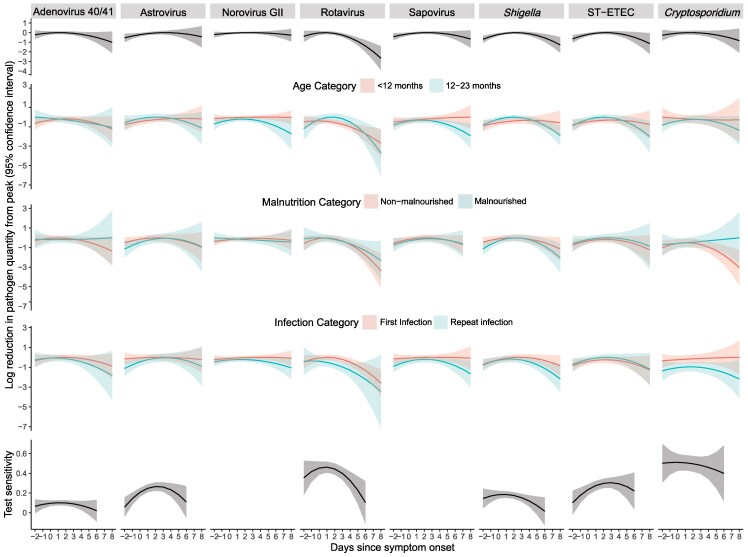
Pathogen log quantity by quantitative PCR (qPCR) and non-qPCR test sensitivity (bottom row) as a function of day of sample collection, where day 0 is the first day of symptoms, during diarrheal episodes for the top 8 causes of diarrhea in the MAL-ED study. ST-ETEC, heat-stable toxin-producing *Escherichia coli.*

Peak pathogen quantity occurred around day 2 (median 2; interquartile range [IQR] 1.5, 2.5) (Figure 1). Rotavirus had a nearly 2-log reduction in pathogen quantity from peak by day 7 (−1.98; 95% confidence interval [CI] −2.88, −1.08) (Table 2). In comparison, *Shigella* (−0.87; −1.46, −.28), adenovirus 40/41 (−0.73; −1.54, .08), and ST-ETEC (−0.78; −1.57, .02) showed nearly a 1-log reduction. Astrovirus (−0.27; −1.07, .54), *Cryptosporidium* (−0.59; −1.51, .33), norovirus GII (−0.18; −.65, .29), and sapovirus (−0.45; −1.10, .20) exhibited smaller reductions.

**Table 2.** Pathogen Log Quantity Reductions (95% Confidence Intervals) From Peak to Day 7 of Symptoms, Both Overall and Stratified by Host Factors, and Absolute Reduction in Original Microbiologic Test Sensitivity From Peak to Day 6

	Adenovirus 40/41	Astrovirus	Norovirus GII^a^	Rotavirus	Sapovirus	*Shigella*	ST-ETEC	*Cryptosporidium*
Overall	−0.73 (−1.54, .08)	−0.27 (−1.07, .54)	−0.18 (−.65, .29)	−1.98 (−2.88, −1.08)	−0.45 (−1.10, .20)	−0.87 (−1.46, −.28)	−0.78 (−1.57, .02)	−0.59 (−1.51, .33)
Age category								
<12 m of age	−0.70 (−1.64, .24)	0.04 (−.98, 1.06)	−0.00 (−.51, .50)	−1.73 (−2.75, −.70)	0.20 (−.67, 1.06)	−0.09 (−1.00, .82)	−0.28 (−1.32, .76)	−0.08 (−1.69, 1.54)
12–23 m of age	−0.71 (−2.15, .73)	−0.75 (−1.97, .46)	−1.03 (−2.18, .13)	−2.61 (−4.30, −.92)	−1.14 (−2.06, −.22)	−1.32 (−2.04, −.59)	−1.39 (−2.55, −.24)	−0.78 (−1.83, .26)
Malnutrition category								
Nonmalnourished	−0.99 (−2.06, .08)	−0.63 (−1.71, .44)	−0.12 (−.78, .54)	−2.48 (−3.73, −1.24)	−0.70 (−1.60, .20)	−0.79 (−1.62, .03)	−0.73 (−1.81, .34)	−1.81 (−3.05, −.58)
Malnourished	0.13 (−1.85, 2.12)	−0.52 (−2.33, 1.29)	−0.17 (−1.16, .81)	−1.74 (−3.15, −.32)	−0.50 (−1.85, .86)	−1.39 (−2.45, −.33)	−0.56 (−2.26, 1.13)	0.37 (−1.49, 2.23)
Infection category								
First infection	−0.61 (−1.50, .28)	−0.15 (−1.09, .78)	−0.03 (−.58, .52)	−1.91 (−2.85, −.98)	−0.03 (−0.86, .80)	−0.53 (−1.25, .18)	−0.70 (−1.74, .34)	0.14 (−1.02, 1.30)
Repeat infection	−1.33 (−3.10, .44)	−0.55 (−1.95, .86)	−0.56 (−1.36, .24)	−2.28 (−4.89, .32)	−1.02 (−1.99, −.05)	−1.43 (−2.37, −.48)	−0.81 (−1.97, .36)	−0.87 (−2.16, .42)
Original diagnostic sensitivity	8.1% (−3.0%, 19.2%)	15.7% (−.4%, 31.8%)	NA	36.0% (14.2%, 57.8%)	NA	17.0% (2.8%, 31.2%)	8.4% (−10.4%, 27.1%)	11.2% (−17.2%, 39.6%)

Malnourished was defined as a length-for-age *Z* score <−2.

Abbreviation: ST-ETEC, heat-stable toxin-producing *Escherichia coli*.

^a^In the original microbiologic work-up, norovirus GII was detected by qPCR and sapovirus was not detected; thus, no conventional test result was available.

Pathogen quantity consistently declined more rapidly in children aged 12–23 months, with statistically significant difference in pathogen kinetics observed for rotavirus, *Shigella*, ST-ETEC, norovirus GII, and sapovirus (Table 2). Rotavirus showed a roughly 2.5-log reduction in pathogen quantity from its peak by day 7 (−2.61; −4.30, −.92) for children aged 12–23 months compared to a roughly 1.5-log reduction (−1.73; −2.75, −.70) for children aged 0–11 months. There was statistically significant difference in pathogen kinetics by chronic malnutrition status for *Cryptosporidium* and *Shigella*. In children without chronic malnutrition, *Cryptosporidium* pathogen quantity decreased by roughly 2 logs (−1.81; −3.05, −.58) from peak quantity to day 7, while for children with chronic malnutrition, no decrease in *Cryptosporidium* quantity was observed (0.37; −1.49, 2.23).

All pathogens except for ST-ETEC exhibited a lower peak pathogen quantity, a sharper decline in pathogen quantity, or both during repeat infections compared to initial infections, with a statistically significant difference in pathogen kinetics between initial and repeat infections observed for astrovirus and sapovirus. No appreciable reduction in quantity was observed from peak to day 7 during initial infection for astrovirus, norovirus GII, sapovirus, or *Cryptosporidium.* For repeat *Cryptosporidium* infections, we estimated a 0.8-log lower peak quantity (−0.78; −1.20, −.36) and a 1.8-log difference in quantity (−1.82; −3.49, −.16) on day 7 in subsequent as compared to initial infections. *Shigella* had a similar peak quantity but declined by 0.5 logs (−0.53; −1.25, .18) during initial infections versus nearly 1.5 logs (−1.43; −2.37, −.48) during subsequent infections.

All pathogens other than norovirus GII and sapovirus had non-PCR diagnostics used in the original microbiologic work-up. Overall test sensitivity was 9.1% for adenovirus, 23.3% for astrovirus, 44.8% for rotavirus, 13.7% for *Shigella*, 22.3% for ST-ETEC, and 45.9% for *Cryptosporidium*. We modeled test sensitivity as a function of symptom duration prior to stool sample collection and observed a peak sensitivity on symptom day 1 (median 1; IQR 1, 2). Rotavirus EIA declined from a peak of 46.2% (40.7, 51.8) on day 1 to 10.2% (−11.6, 32.0) by day 6, an absolute reduction of 36.0% (Table 2). *Shigella* culture sensitivity declined from a peak of 18.5% (15.2, 21.8) on day 1 to 1.5% (−12.7, 15.7) by day 6, an absolute reduction of 17.0%. Total attribution by qPCR increased from 21.7% 2 days prior to symptom onset and 34.3% 1 day prior to symptom onset to a peak of 50.9% on the day of symptom onset and 48.5% on the second day of symptoms. It then declined roughly linearly to 33.0% on day 5, after which it was below 30%.

## DISCUSSION

Using data from the MAL-ED birth cohort study, we found that peak quantity consistently occurred during the first few days of symptoms, but that kinetics were pathogen specific. For instance, rotavirus quantity had a 2-log decline by day 7, whereas adenovirus 40/41, ST-ETEC), and *Shigella* had near 1-log declines, and astrovirus, *Cryptosporidium*, norovirus GII, and sapovirus exhibited negligible change. These findings underscore the importance of collecting stool samples early in the illness to maximize the accuracy of etiology assignment, as a rapid decline in detectable pathogen quantity, for example for rotavirus, may lead to an underestimation of the disease burden. The findings also suggest that existing etiology estimates could be biased toward pathogens that maintain a higher quantity in stool for a longer period of time, as they are more likely to be detected by non-qPCR methods and more likely to be detected at high quantities by qPCR. Notably, this means that rotavirus and *Shigella* are typically the top 2 etiologies of diarrhea in studies using molecular methods despite this possible diagnostic bias.

These findings have implications for studies where pathogen-specific quantitative cutoffs are applied, for example, diarrhea treatment trials [4] or vaccine efficacy trials [5], as children who present later in illness are less likely to have an etiology identified. For efficacy trials with passive surveillance for outcomes, children should present to study facilities as close to symptom onset as possible. The tolerances around the optimal day of sample collection are also pathogen specific. Importantly, with the exception of *Cryptosporidium*, the peak pathogen quantity appeared to be independent of child age and nutritional status and was similar between initial and subsequent infections. This suggests that the sensitivity of pathogen detection and assignment of etiology by qPCR when samples are collected early in illness should also not be modified by these host factors.

In a previous analysis of the postdiarrheal duration of pathogen detection using MAL-ED data [6], we found rotavirus to have the shortest detection duration and *Cryptosporidium*, norovirus, and sapovirus the longest. The pathogen kinetics described in the current analysis are consistent with those findings. Our results are also consistent with previous single-pathogen analyses. In a small set of children in India, peak rotavirus quantity occurred early in infection and declined rapidly during the first week [8]. Similarly, ETEC declined rapidly from a peak several days after challenge in an adult challenge model [9], while prolonged detection of norovirus was observed in an adult challenge model [10].

Our results also have implications for the use of non-PCR tests. For rotavirus, EIA sensitivity was high at symptom onset but declined rapidly in parallel with qPCR quantity, whereas for *Cryptosporidium*, EIA sensitivity was more resilient to the day of sample collection. Our findings are consistent with the re-analysis of the Global Enteric Multicentre Study, which found that rotavirus and *Cryptosporidium* had the best sensitivity among non-PCR tests [5]. However, our study is additionally able to describe the significant effect that sample collection timing can have on diagnostic sensitivity, such that rotavirus EIA sensitivity changed rapidly over the first week of illness, whereas *Cryptosporidium* EIA sensitivity did not. Interestingly, the peak sensitivity of the original microbiologic tests, mostly EIA and bacterial culture, was on average earlier in disease than was the peak quantity by qPCR. This might suggest that non-PCR test sensitivity is a function of more than just pathogen quantity. In particular, it is likely driven by pathogen viability, both for successful growth in culture as well as for detection of intact expressed proteins.

Several limitations should be considered. First, we did not have serial samples and instead relied on the natural distribution of sample collection in the MAL-ED cohort. This collection was therefore biased toward the early days of symptoms, reducing precision for the later stages of infection. We thus also could not conclusively determine the exact duration of pathogen shedding for each episode. Additionally, we could not limit our analysis to high-quantity detections, which may be most likely to be etiologic, without losing the kinetic perspective. Therefore, we considered an episode likely etiologic if a pathogen was detected and no other etiology was identified. However, the use of a well-characterized longitudinal cohort with a daily symptom history permitted the precise modeling of the relationship between symptom day and pathogen quantity as well as the evaluation of effect modifiers, including prior infection and malnutrition. Finally, all analyses were conducted within-pathogen; however, differences in gene targets and amplicon sizes may have resulted in minor variability in amplification efficiency across qPCR assays, which could affect cross-pathogen comparisons.

These analyses add a temporal dimension to our understanding of diagnostic tests performance for diarrheal disease. In particular, they highlight that collection timing may significantly alter etiology attribution for both qPCR and traditional tests. Additionally, they demonstrate that test performance may be more sensitive to the day of collection for some pathogens (eg, rotavirus) compared to others (eg, norovirus and *Cryptosporidium*) and that viability-dependent tests are particularly vulnerable to collection delays. To optimally reduce the bias introduced by these kinetics, studies where etiology assignment is critical, including treatment and vaccine trials, should consider strict collection windows early in disease for sample collection whenever feasible.
